# Decreased renal or cerebral oxygen saturation increases the risk of cardiac surgery-associated acute kidney injury in adult patients: a prospective observational study

**DOI:** 10.1186/s12871-025-03296-4

**Published:** 2025-08-29

**Authors:** Bo Wang, Hui Shi, Hua Zhang, Yongjie Chen, Shen Liu, Bin Li, Chunxia Shi

**Affiliations:** 1 Department of Anesthesiology, Peking University International Hospital, No.1, Shengmingyuan Road, Changping District, Beijing, China; 2 Department of Anesthesiology, Shijingshan Teaching Hospital of Capital Medical University, Beijing Shijingshan Hospital, Beijing, China; 3 Research Center of Clinical Epidemiology, Peking University Third Hospital, Beijing, China; 4 Department of Cardiac Surgery, Peking University People’s Hospital, Beijing, China; 5 Department of Cardiac Surgery, Peking University International Hospital, Beijing, China

**Keywords:** Acute kidney injury, Cardiac surgery, Near-infrared spectroscopy, Renal oxygen saturation, Cerebral oxygen saturation

## Abstract

**Objective:**

To determine the relationship between intraoperative decreased renal and cerebral oxygen saturation, as measured using near-infrared spectroscopy (NIRS), and cardiac surgery-associated acute kidney injury (CSA-AKI) in adults.

**Methods:**

This prospective observational study was conducted at Peking University International Hospital. Between November 2022 to August 2023, 101 adult patients undergoing cardiac surgery under cardiopulmonary bypass were included. Renal and cerebral tissue oxygen saturation was continuously monitored during the operation using NIRS.

**Results:**

The overall incidence of CSA-AKI was 27% (27/101), with a 4% incidence rate of requiring renal replacement therapy. The incidence of CSA-AKI was 57% (13/23) in patients with renal desaturation compared to 18% (14/78) in those without renal desaturation (*P* < 0.01). CSA-AKI occurred in 71% (12/17) of patients with cerebral desaturation compared to 18% (15/84) in those without cerebral desaturation (*P* < 0.01). The incidence of CSA-AKI was 100% (7/7) in patients with simultaneous renal and cerebral desaturation. Renal desaturation alone showed a sensitivity of 48%, while the combination of renal and cerebral desaturation demonstrated 100% specificity for predicting CSA-AKI.

**Conclusions:**

In adult patients, 27% experience CSA-AKI. Intraoperative renal or cerebral desaturation, as monitored by NIRS, is associated with a higher risk of CSA-AKI, with simultaneous renal and cerebral desaturation which yielded the highest specificity in predicting postoperative AKI.

**Trial registration:**

This study has been registered on the Chinese Clinical Trial Registry number (ChiCTR2200065161, 30/10/2022).

## Introduction

Cardiac surgery-associated acute kidney injury (CSA-AKI) not only increases the medical burden but also raises patient mortality [1]. For patients with severe AKI, there are no effective treatments besides supportive care and dialysis [2]. Although changes in serum creatinine are the primary basis for diagnosing AKI, they lack sensitivity, as nearly 50% of the glomerular filtration rate (GFR) must be lost in healthy individuals before changes in serum creatinine can be detected [3]. Therefore, there is a need to actively seek methods for early detection of AKI. Additionally, serum biomarker testing and continuous urine output monitoring are inadequate for real-time intraoperative renal function assessment, which is critical for optimizing renal outcomes.

The exact mechanism of postoperative AKI is unclear and is associated with multiple factors, all of which share the characteristic of renal oxygen supply being lower than oxygen consumption [4]. During cardiopulmonary bypass, reduced renal blood flow leads to renal medullary hypoxia, which subsequently causes a decline in GFR. Enhancing renal blood flow and improving medullary perfusion demonstrate renal protective effects [5, 6]. Research has demonstrated that the degree of renal medullary hypoxia correlates with urinary oxygen concentration (PuO2), and decreased PuO2 is associated with postoperative AKI occurrence [7]. Urinary oxygen concentration serves as an indirect indicator of renal oxygenation status, while the advent of near-infrared spectroscopy (NIRS) enables direct and continuous monitoring of renal oxygenation. NIRS is a continuous and real-time monitoring tool that reflects regional tissue oxygen saturation (rSO2). It measures rSO2 based on the relative concentrations of oxyhemoglobin and deoxyhemoglobin in the tissue [8]. Tissue oxygen saturation represents the balance between oxygen supply and consumption, with a decrease in oxygen saturation indicating insufficient tissue perfusion [9].

Both the brain and kidneys have the ability to autoregulate blood flow, maintaining normal tissue perfusion when blood pressure changes [10, 11]. Although the brain is less tolerant to ischemia, the kidney’s autoregulatory capacity is not as robust as that of the brain. When cerebral perfusion is insufficient, the kidneys may already be experiencing hypoxia. Recent studies have found that the brain, as an “indicator organ” of overall oxygen sup-ply-demand balance, shows that a decrease in cerebral oxygen saturation is closely related to postoperative AKI, serving as an early marker of kidney injury [12]. Direct monitoring of renal rSO2 in adults using NIRS has revealed [13, 14] that a decline in renal rSO2 is associated with postoperative AKI, suggesting that NIRS monitoring of renal rSO2 and perfusion could be a potential predictor of AKI. Given the limited penetration depth of NIRS, there is more research on renal rSO2 monitoring in infants and young children than in adults.

In this prospective observational study, we investigated the relationship between intraoperative renal and cerebral rSO2 and CSA-AKI in adult patients undergoing cardiopulmonary bypass (CPB). We hypothesize that decreased renal and cerebral rSO2 indicates insufficient renal tissue perfusion, which is associated with CSA-AKI.

## Methods

### Study design and population

**Fig. 1:**
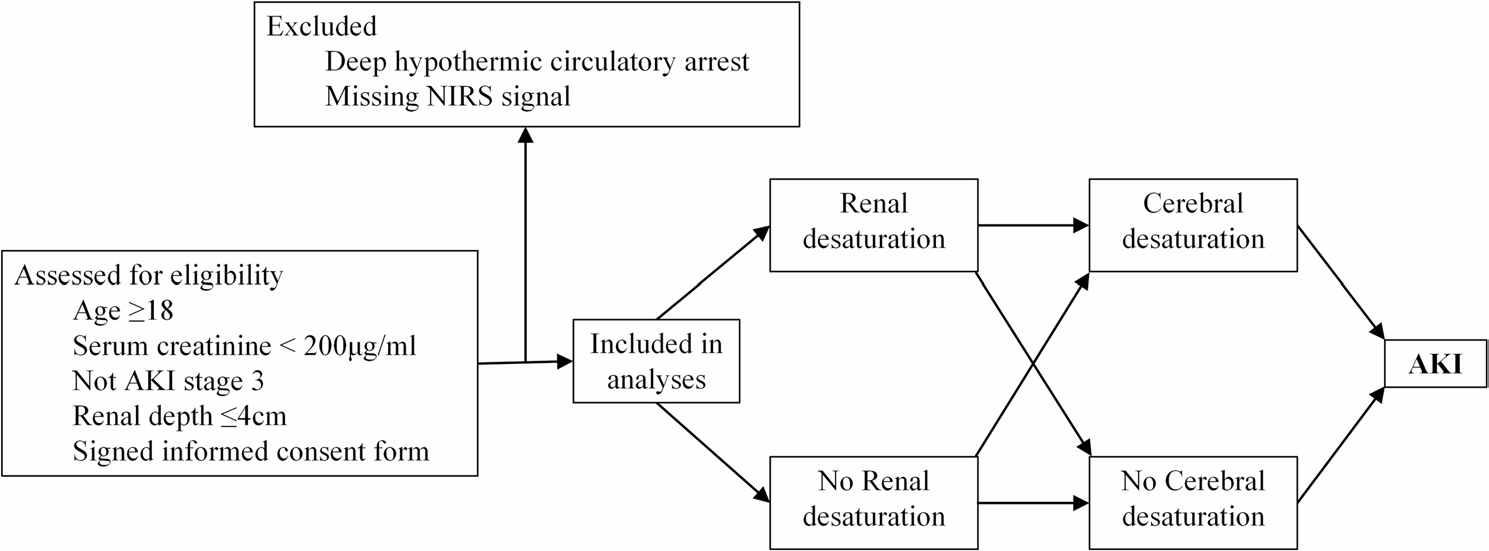
Patient enrollment flowchart. AKI, acute kidney injury

This prospective observational cohort study was conducted at Peking University International Hospital. Patient enrollment took place from November 2022 to August 2023. The study protocol was approved by the Biomedical Ethics Committee of Peking University International Hospital (2022-KY-0012-02). All eligible subjects or their legally authorized representatives provided written informed consent. Inclusion criteria: adult patients (≥ 18 years) scheduled for cardiac surgery under CPB, preoperative serum creatinine < 200 µg/ml, not diagnosed AKI stage 3, renal depth (distance from the skin surface to the kidney) < 4 cm. Exclusion criteria: deep hypothermic circulatory arrest (DHCA), or intraoperative loss of NIRS signal. Refer to Fig. 1.

### Near-infrared spectroscopy

Tissue oxygenation was monitored using the NIRS monitor (INVOS 5000 C, Medtronic, Minneapolis, Minnesota, USA). Before anesthesia induction, the depth of the left kidney was measured using ultrasound. If the kidney depth was < 4 cm, the NIRS electrodes (SAFB-SM/INTL20) were placed in the left renal area and the left frontal position. Otherwise, the patient was excluded. Before any drug administration, baseline values for renal and cerebral oxygen saturation were recorded in an air-breathing state (T0). The remaining 10 recording time points are: endotracheal intubation (T1), skin incision (T2), after heparinization (T3), ascending aorta cannulation(T4), 10 min after CPB (T5), 10 min after rewarming (T6), 5 min after opening the ascending aorta (T7), 20 min after the end of CPB (T8), after chest closure (T9), and at the end of the surgery (T10). Renal and cerebral tissue desaturation was defined as a decrease of at least 20% compared to baseline (T0) at any one or more sampling time points. If the NIRS signal was absent, the patient would be excluded from the analysis.

### Outcomes

The primary outcome was the incidence of CSA-AKI, assessed according to the Kidney Disease: Improving Global Outcomes (KDIGO), defined as an increase in serum creatinine of ≥ 26.5 µmol/L within 48 h or an increase in serum creatinine to > 1.5 times the baseline value within 7 days. The baseline value is defined as the most recent measurement obtained within 7 days before surgery. Due to its unreliability, urine output was not included in the AKI assessment. Secondary outcomes included the duration of postoperative mechanical ventilation (time from CCU admission to extubation), length of stay in the CCU (time from CCU admission to CCU discharge), in-hospital mortality (death during hospitalization), length of post-operative hospital stay (time from the first postoperative day to discharge), total hospital length of stay (time from admission to discharge), and adverse events within 30 days post-surgery. The relationship between renal rSO2 and non-renal outcomes was also explored.

### Statistical analysis

Continuous variables that follow a normal distribution are expressed as mean ± standard deviation and analyzed using Student’s t-test for independent samples. Continuous variables with skewed distributions are expressed as median with interquartile range and analyzed using the Mann-Whitney U test. Categorical variables are presented as counts and percentages and analyzed using Fisher’s exact test. The correlation between cerebral and renal rSO2 is assessed using Spearman correlation coefficients. Sensitivity, specificity, and the Youden index were used to assess the diagnostic value of cerebral and renal oxygen desaturation for AKI. The formula for the Youden index is: Sensitivity + Specificity − 1.

## Results

### Patient characteristics

Between November 2022 and August 2023, a total of 317 patients were screened to determine eligibility. Ultimately, 101 patients were included in the analysis. Among these, 23 patients (23%) experienced at least one episode of reduced renal rSO2 during surgery and 7 patients (7%) experienced simultaneous reductions in both cerebral and renal rSO2. Cerebral desaturation occurred in 17 patients (17%), including patients with and without renal desaturation. Compared with the group without renal desaturation, no significant differences were found in the group with renal desaturation except for a significantly higher proportion of patients undergoing re-do surgeries demonstrated in Table 1.

**Table 1:** Baseline and demographic characteristics

	Overall (*n* = 101)	Without renal desaturation (*n* = 78)	With renal desaturation (*n* = 23)	*P*
Age(years)	52(32–64)	52(31–62)	54(33–67)	0.232
Male, n(%)	44(44)	34(44)	10(44)	1.000
BMI (kg/m^2^)	21.5 ± 2.8	21.5 ± 2.8	21.2 ± 2.8	0.657
Re-do surgery, n(%)	26(26)	15(19)	11(48)	0.013
Disease, n(%)				0.613
VHD				
Single	13(13)	11(14)	2(9)	
Double/multiple	28(28)	19(24)	9(39)	
CAD	21(21)	17(22)	4(17)	
CP	6(6)	4(5)	2(9)	
CHD	33(33)	27(35)	6(26)	
Comorbidity, n(%)				
Hypertension	21(21)	17(22)	4(17)	0.776
Diabetes	18(18)	15(19)	3(13)	0.757
Hyperlipidemia	13(13)	10(13)	3(13)	1.000
CKD	16(16)	11(14)	5(22)	0.515
Heart failure	35(35)	28(36)	7(30)	0.804
Arrhythmia	31(31)	22(28)	9(39)	0.318
PH	40(41)	34(44)	6(26)	0.152
NYHA class				0.498
I	33	23	10	
II	35	29	6	
III	23	19	4	
IV	10	7	3	
Creatinine(µmol/L)	74(65–86)	74(65–84)	72(63–93)	0.997
Cystatin C (mg/L)	1.0(0.9–1.4)	1.0(0.8–1.3)	1.1(0.9–1.4)	0.339
eGFR(ml/min/1.73m^2^)	90.1 ± 25.5	91.0 ± 25.7	87.1 ± 25.1	0.530
Renal depth (cm)	3.3(2.8–3.7)	3.3(2.7–3.7)	3.4(3.0-3.7)	0.814

*BMI* Body mass index, *CAD* Coronary atherosclerotic heart disease, *CHD* Congenital heart disease, *CKD* Chronic kidney disease, *CP* Constrictive pericarditis, *eGFR* Estimated glomerular filtration rate, *NYHA* New York Heart Association, *PH* Pulmonary hypertension, *VHD* Valvular heart disease

### Renal rSO2 and outcomes

As shown in Table 2, the overall incidence of postoperative AKI was 27% (27/101). Among these, 20 patients had KDIGO stage 1 AKI, 3 patients had stage 2 AKI, and 4 patients had stage 3 AKI and required dialysis. Of the patients who developed AKI, 14 were diagnosed on postoperative day 1, 11 on postoperative day 2, and 2 on postoperative day 3. There were no deaths in the group without renal desaturation during hospitalization, whereas 3 patients died in the group with renal desaturation. Compared to the group without renal desaturation, the renal desaturation group had significantly longer aortic clamping(*P* = 0.002) and CPB time(*P* < 0.001), prolonged surgical(*P* < 0.001) and mechanical ventilation durations(*P* = 0.004), as well as extended CCU(*P* = 0.004) and postoperative hospital stays(*P* = 0.029). There was no statistical difference in the proportion of patients with reduced cerebral oxygen saturation between the two groups. Although lactate levels were higher in the renal desaturation group during and immediately after CPB(*P* = 0.002), no significant differences were observed between the groups in the later postoperative period(*P* = 0.513). Similarly, although hematocrit was lower in the renal desaturation group after CPB(*P* = 0.038), the two groups showed no significant differences postoperatively in hematocrit levels(*P* = 0.133). Comparing preoperative and postoperative kidney function indicators (Table 3), patients in the renal desaturation group had higher postoperative creatinine and cystatin C levels and lower GFR.

**Table 2:** Intraoperative characteristics and outcomes

	Overall (*n* = 101)	Without renal desaturation (*n* = 78)	With renal desaturation (*n* = 23)	*P*
Baseline renal rSO_2_(%)	86(79–89)	85(78–89)	87(82–89)	0.265
Baseline cerebral rSO_2_(%)	63 ± 9	63 ± 10	62 ± 9	0.776
*Intraoperative*				
Cerebral rSO_2_ decline from baseline>20%, n(%)	17(17)	10(13)	7(30)	0.060
Urine volume (ml)				
Before CPB	120(50–300)	120(50–300)	100(50–300)	0.830
CPB	410(220–1055)	390(200–895)	500(300–1200)	0.307
After CPB	350(200–590)	400(200–600)	300(160–500)	0.314
Total	1120(700–1870)	1080(665–1950)	1350(760–1800)	0.590
Lac (mmol/L)				
Before CPB	0.8(0.6–0.9)	0.8(0.6–0.9)	0.8(0.7–0.9)	0.211
CPB	1.1(0.8–1.5)	1.1(0.8–1.3)	1.5(1.0-1.8)	0.002
After CPB	1.6(1.0-2.4)	1.5(1.0-2.2)	2.4(1.5–3.3)	0.002
Hct (%)				
Before CPB	38(35–42)	38(35–42)	37(35–40)	0.436
CPB	27(25–30)	27(25–30)	29(25–31)	0.868
After CPB	31(28–33)	32(28–34)	29(28–31)	0.038
Aortic cross clamp (min)	109(73–166)	97(70–153)	150(125–176)	0.002
CPB time(min)	159(111–224)	137(100–209)	218(180–283)	0.000
Surgery time(min)	304(237–420)	271(229–370)	420(310–495)	0.000
*Postoperative*				
Lac (mmol/L)	1.9(1.3–2.8)	1.9(1.2–2.9)	2.1(1.5–2.6)	0.513
Hct (%)	34(31–38)	34(31–39)	33(30–36)	0.133
Ventilation(h)*	18(16–20)	18(15–20)	21(18–42)	0.004
CCU time(d)*	2(2–3)	2(2–3)	4(2–5)	0.004
Postoperative AKI, n(%)				0.000
None^#^	74(73)	64(82)	10(44)	
Stage 1	20(20)	14(18)	6(26)	
Stage 2^#^	3(3)	0(0)	3(13)	
Stage 3^#^	4(4)	0(0)	4(17)	
In-hospital mortality, n(%)	3(3)	0(0)	3(13)	0.011
Length of postoperative hospital stay(d)*	9(7–12)	8(7–12)	10(8–19)	0.029
Total hospital length of stay(d)*	17(13–22)	16(13–21)	18(13–26)	0.397

*Patients who died were not included. #After multiple comparisons, there were differences between the two groups

*rSO*_2_ Regional tissue oxygen saturation, *CPB* Cardiopulmonary bypass, *Lac* Lactic acid, *Hct* Hematocrit, *CCU* Cardiac intensive care unit, *AKI* Acute kidney injury, *h* hour, *d* day

**Table 3:** Changes of preoperative and postoperative kidney function indicators

	Overall (*n* = 101)	Without renal desaturation (*n* = 78)	With renal desaturation (*n* = 23)	*P*
Creatinine(µmol/L)				
Before op	74(65–86)	74(65–84)	72(63–93)	0.997
Post-op 24 h	78(66–102)	76(64–92)	88(78–130)	0.002
Post-op 48 h	75(63–95)	73(61–87)	99(75–147)	0.002
Post-op 72 h	75(63–98)	72(61–93)	95(71–150)	0.004
Cystatin C (mg/L)				
Before op	1.0(0.9–1.4)	1.0(0.8–1.3)	1.1(0.9–1.4)	0.339
Post-op 24 h	0.9(0.8–1.4)	0.9(0.8–1.1)	1.1(0.9–1.7)	0.009
Post-op 48 h	1.1(0.9–1.6)	1.0(0.8–1.3)	1.4(1.1-2.0)	0.001
Post-op 72 h	1.1(0.9–1.6)	1.1(0.8–1.5)	1.4(1.1–1.8)	0.004
eGFR(ml/min/1.73m^2^)				
Before op	90.1 ± 25.5	91.0 ± 25.7	87.1 ± 25.1	0.530
Post-op 24 h	82.1 ± 27.4	86.7 ± 26.3	66.7 ± 25.8	0.002
Post-op 48 h	84.3 ± 28.4	89.3 ± 25.8	67.3 ± 31.0	0.001
Post-op 72 h	84.8 ± 27.8	89.6 ± 25.2	68.330.6	0.001

*eGFR* Estimated glomerular filtration rate, *op* Operation

### Renal and cerebral rSO2

**Fig. 2:**
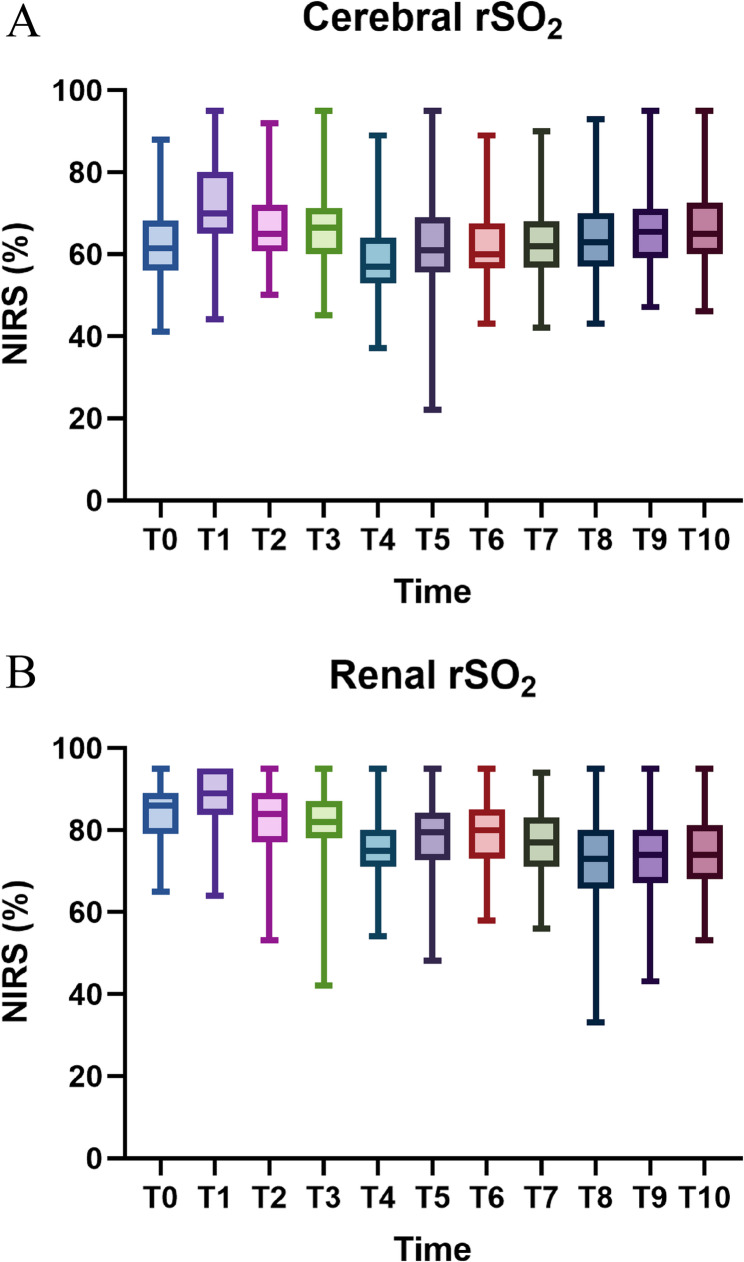
Box plot of cerebral and renal rSO2 values for all patients at different time points: baseline values(T0), endotracheal intubation(T1), skin incision (T2), after heparinization (T3), ascending aorta cannulation(T4), 10 minutes after CPB (T5), 10 minutes after rewarming (T6), 5 minutes after opening the ascending aorta (T7), 20 minutes after the end of CPB (T8), after chest closure (T9), and at the end of the surgery (T10)

**Fig. 3:**
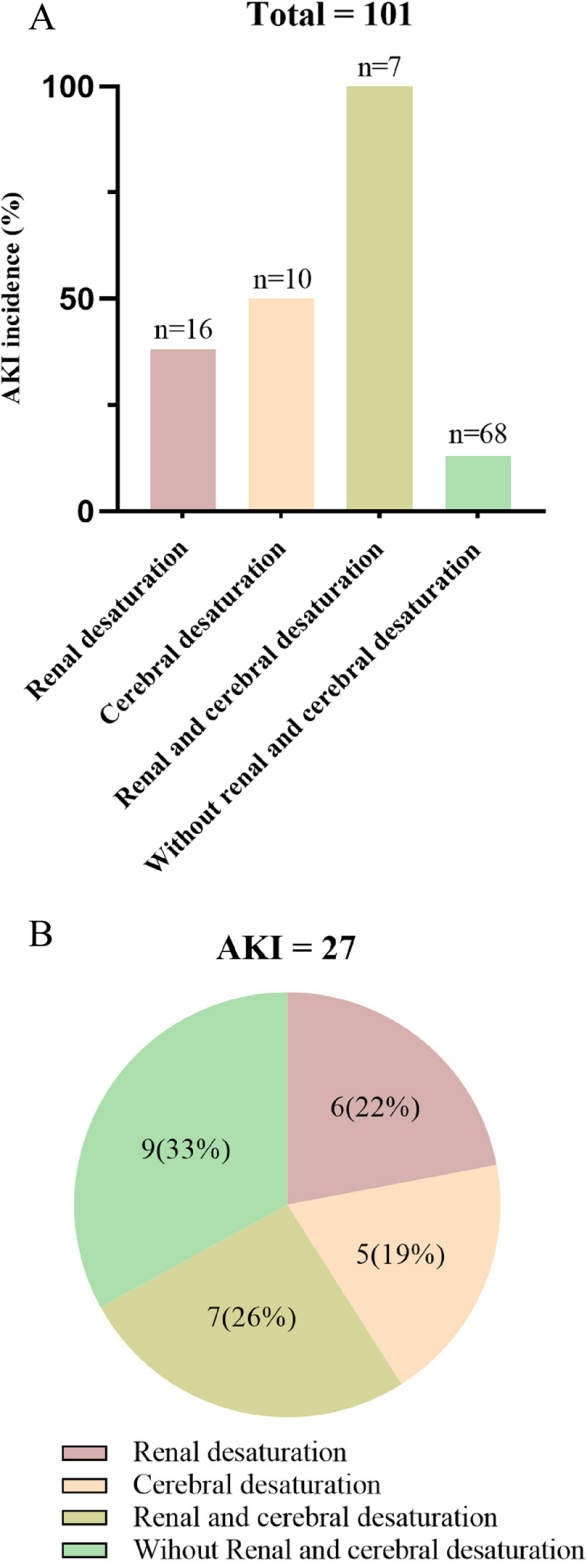
Renal desaturation, cerebral desaturation and acute kidney injury (AKI). **A** Incidence of AKI in patients with renal desaturation, cerebral desaturation, both renal and cerebral desaturation, and neither renal nor cerebral desaturation. **B** Numbers (proportions) of AKI identified by renal desaturation, cerebral desaturation, both renal and cerebral desaturation, and neither renal nor cerebral desaturation

The changes in renal and cerebral rSO2 at different time points for all patients are shown in Fig. 2. Spearman correlation analysis revealed a positive correlation between renal and cerebral rSO2 (*r* = 0.356, *P* < 0.001). The incidence of AKI was 57% (13/23) in patients with reduced renal rSO2 and 71% (12/17) in those with reduced cerebral rSO2, compared to 18% (14/78, 15/84) in patients without desaturation in either region (*P* < 0.01 for both). AKI occurred in 38% (6/16) of patients with isolated renal desaturation, 50% (5/10) with isolated cerebral desaturation, and 100% (7/7) of those with both. In contrast, only 13% (9/68) of patients without any desaturation developed AKI (Fig. 3 A). The distribution of desaturation patterns among the 27 AKI patients is shown in Fig. 3B. The sensitivity, specificity, and Youden index for isolated reduced cerebral rSO2, isolated reduced renal rSO2, and simultaneous reductions in both cerebral and renal rSO2 are summarized in Table 4.

**Table 4:** Sensitivity and specificity and Youden index of renal and cerebral desaturation in identifying postoperative acute kidney injury

	Sensitivity	Specificity	Youden Index
Renal desaturation	0.48(95%CI:0.29, 0.67)	0.86(95%CI:0.79, 0.94)	0.35
Cerebral desaturation	0.44(95% CI:0.26,0.63)	0.93(95%CI:0.88,0.99)	0.38
Renal and Cerebral desaturation	0.26(95%CI:0.09,0.42)	1(95%CI:1,1)	0.26

## Discussion

This study found that the incidence of CSA-AKI in adult patients was 27%, with a 4% incidence rate of requiring renal replacement therapy, which is consistent with previous reports [1]. Patients with reduced renal or cerebral rSO2 during surgery had a higher risk of developing AKI postoperatively, particularly those with simultaneous reductions in both renal and cerebral rSO2. Monitoring rSO2 using NIRS may help in the early pre-diction of kidney function impairment, allowing for prompt intervention and treatment.

NIRS technology was primarily used to monitor cerebral oxygenation during the perioperative period. Due to the brain’s autoregulatory mechanisms for blood flow, appropriate perfusion pressure is necessary to ensure adequate cerebral perfusion and NIRS values. The brain can act as an “indicator organ,” optimizing mean arterial pressure to ensure cerebral perfusion while benefiting other organs’ functions [15, 16]. Similarly, renal blood flow is autoregulated, and maintaining adequate cerebral oxygen saturation can ensure sufficient renal perfusion [17, 18]. Studies have found that cerebral rSO2 values are lower in CSA-AKI patients compared to those without AKI [19], and the variability in rSO2 may have higher predictive capability [20]. In non-cardiopulmonary bypass patients, a lower average cerebral rSO2 during surgery is also independently associated with postoperative AKI [12]. In this study, 17 patients had more than a 20% reduction in cerebral oxygenation during surgery. The proportion of patients with reduced cerebral oxygenation was higher in the renal desaturation group (30% vs. 13%), and the incidence of AKI was significantly higher in the reduced cerebral rSO2 group compared to the non-reduction group (71% vs. 18%, *P* < 0.01), supporting the association between reduced cerebral oxygenation and AKI.

NIRS technology was initially applied to monitor renal oxygen saturation in children to reduce the incidence of AKI and improve renal outcomes, particularly in infants and young children. Studies have shown a significant correlation between AKI and low renal oxygen saturation in premature infants [21–23]. Children who develop AKI after cardiac surgery may experience varying degrees of low renal oxygen saturation during different periods of surgery or the postoperative period [24–28], with decreasing levels correlating with the severity of AKI [29]. Tholen et al. found that for adult patients with a renal depth of no more than 4 cm, NIRS-measured renal rSO2 correlated well with invasive measurements of renal venous oxygen saturation (SrvO2) and showed minimal deviation [30]. Excluding adult patients with a renal depth greater than 4 cm, Choi et al. found that among patients undergoing cardiac surgery who developed postoperative AKI, the total time during which renal oxygen saturation was below a specific threshold was significantly longer compared to patients who did not develop AKI, with rSO2 < 55% providing the best predictive effect [31]. Ortega-Loubon et al. recommended that postoperative renal rSO2 should not fall below 65% or decrease more than 20% from baseline to prevent CSA-AKI [13]. Recent studies in non-cardiac surgical adult patients also found a significant correlation between intraoperative reductions in renal oxygen saturation and an increased risk of postoperative AKI [14], with a “high-low” trajectory of renal oxygen saturation [32]. In this study, 23 patients had more than a 20% reduction in renal oxygen saturation during surgery, and their incidence of AKI was significantly higher compared to those without reduced renal oxygen saturation (57% vs. 18%, *P* < 0.01), with a higher mortality rate. The patients who died had longer postoperative treatment times, severely affecting postoperative indicators. Even excluding deceased patients, the reduced renal rSO2 group had significantly longer postoperative ventilation times, CCU stays, and discharge times compared to the non-reduced renal oxygen group.

The mechanism of CSA-AKI is complex, but a common feature is that the oxygen supply to the kidneys is insufficient to meet their oxygen demand, leading to tubular damage [4]. CPB can induce renal vascular constriction and renal blood flow redistribution [33], and the duration of CPB and aortic clamping is significantly associated with AKI [34]. Re-do surgeries are also a high-risk factor for CSA-AKI [2]. This study found that the group with renal desaturation had a higher proportion of re-do surgery patients and longer durations of CPB and aortic clamping, which aligns with previous research. Therefore, for patients undergoing re-do heart surgery, it is important to investigate whether the incidence of postoperative AKI can be reduced by minimizing extracorporeal circulation and aortic clamping times.

Elevated lactate levels indicate inadequate tissue perfusion, with both renal desaturation group and the non-desaturation group showing significantly higher lactate levels during and after CPB compared to before. The difference between the groups was statistically significant. Comparing laboratory tests, the preoperative serum creatinine levels were similar between the renal oxygen reduction and non-reduction groups. However, the renal oxygen reduction group had significantly higher creatinine levels at 24, 48, and 72 h postoperatively. Although creatinine is the current gold standard for diagnosing AKI, its accuracy can be influenced by various factors. Therefore, we also compared changes in cystatin C and GFR between the two groups. Similarly, the renal desaturation group had higher levels of cystatin C and lower GFR, which further supports the association between reduced renal rSO2 and renal function impairment.

Spearman correlation analysis revealed a positive correlation between cerebral and renal rSO2, with a positive r value indicating that reductions in cerebral rSO2 are often accompanied by reductions in renal rSO2. However, the small r value suggests that the correlation is not strong, highlighting the importance of localized renal oxygen monitoring. Comparing sensitivity, specificity, and Youden index, we found that the sensitivity of detecting reduced renal rSO2 was the highest, while the specificity of simultaneous reductions in both cerebral and renal rSO2 was the highest. Although the predictive specificity is high, the small sample size of the subgroup necessitates cautious interpretation. The Youden index was highest for reduced cerebral rSO2. Notably, the specificity for simultaneous reductions in both cerebral and renal rSO2 was 1. Although this may be influenced by the relatively small number of patients, it suggests that simultaneous reductions in both cerebral and renal often indicate severe perfusion insufficiency and should be avoided when possible. All three methods demonstrated low sensitivity and cannot accurately predict AKI on their own. However, to reduce the incidence of AKI, efforts should be made to avoid intraoperative reductions in cerebral or renal rSO2 of more than 20%, particularly avoiding simultaneous reductions in both.

This study has several limitations. Firstly, renal rSO2 values are susceptible to positional changes, and sweating can reduce sensor adhesion. Secondly, the normal, pathological, and threshold values for cerebral and renal NIRS are not well established, with significant individual variability. Thirdly, we only monitored renal and cerebral rSO2 during surgery, and the changes in these values postoperatively are not known. Lastly, due to the limited penetration depth of NIRS, renal oxygen saturation monitoring is not feasible for obese patients and those with a kidney depth exceeding 4 cm, which restricts its clinical applicability and generalizability.

## Conclusions

In adult patients undergoing cardiac surgery, 27% experience CSA-AKI. Re-do surgery, prolonged aortic cross clamp time, prolonged CPB time, and prolonged surgery time were identified as high-risk factors for renal desaturation. Intraoperative reductions in renal and cerebral rSO2, as monitored by NIRS, were associated with a higher risk of CSA-AKI. Notably, simultaneous desaturation in both renal and cerebral regions provided the highest specificity for predicting CSA-AKI. Further large-scale studies are warranted to validate these findings.

## Data Availability

The datasets used and analysed during the current study are available from the corresponding author on reasonable request.

## Abbreviations

AKI: Acute kidney injury
CCU: Cardiac intensive care unit
CPB: Cardiopulmonary bypass
CSA-AKI: Cardiac surgery-associated acute kidney injury
GFR: Glomerular filtration rate
KDIGO: Kidney Disease: Improving Global Outcomes
NIRS: Near-infrared spectroscopy
